# Short-Term Effectiveness of the Youth Gambling Prevention Program “Who Really Wins?”—Results from the First National Implementation

**DOI:** 10.3390/ijerph181910100

**Published:** 2021-09-26

**Authors:** Dora Dodig Hundric, Sabina Mandic, Neven Ricijas

**Affiliations:** Department of Behavioral Disorders, Faculty of Education and Rehabilitation Sciences, University of Zagreb, 10 000 Zagreb, Croatia; sabina.mandic@erf.unizg.hr (S.M.); neven.ricijas@erf.unizg.hr (N.R.)

**Keywords:** adolescents, youth gambling, prevention, evaluation, implementation

## Abstract

As a response to significant adolescent gambling involvement, a Croatian team of researchers and practitioners developed a universal, comprehensive, evidence-based youth gambling prevention program called “Who Really Wins?”. This study presents the results on its short-term effectiveness following the first national implementation in 18 Croatian cities, with a total of 629 high school students (66.5% male) who completed the program. A design with two measurement sessions (pre-test and post-test) was used to explore the short-term effects of the program on gambling-related knowledge and cognition, frequency of gambling, and various socio-emotional skills. The results showed significant effects when it comes to knowledge, cognitive distortions, and the frequency of sports betting and playing lottery games. Furthermore, the program had no harmful effects on any of the measured variables. The results are interpreted in terms of methodological challenges in measuring effects, possible improvements of the program, and implications for future evaluation research.

## 1. Introduction

The gambling industry is one of the fastest growing and most pervasive industries in the world [1,2,3]; therefore, it is not surprising that the prevalence of gambling and gambling-related problems are increasing [4,5,6,7,8,9,10,11], particularly in countries with a liberal gambling market [12,13,14,15]. Accordingly, rates of problem gambling in the adult population are significant, ranging from 0.12% to 3.4% in Europe and from 0.12% to 5.8% worldwide [16], but research continuously shows that special attention should be paid to the adolescent population. Due to their developmental characteristics (tendency of risk-taking behaviour, short-term planning, identity formation, resistance to authority, increased reward-seeking, etc.) [13,17,18,19], they stand out as a particular risk group for developing adverse gambling-related consequences, such as mental health or family problems, school problems, disturbed interpersonal relationships, compulsive gambling behaviour, and, the most serious consequence, the development of a gambling addiction [13,20,21,22,23,24,25,26,27,28,29,30].

The latter is supported by a large body of research showing that young people participate in gambling at even higher rates and develop more gambling-related problems compared to adults [26,31,32,33,34]. A recent systematic review of 44 global studies found that from 0.2% to as many as 12.3% of adolescents meet the criteria for problem gambling, with Croatia leading in the prevalence of gambling-related problems [34]. The rate of adolescent problem gambling in Croatia increases up to 23.7% when a subsample of male high school students is analysed [13], reconfirming boys as a population at greater risk for developing gambling-related problems [22,25,29,35,36,37,38]. The high gambling participation of Croatian adolescents is also confirmed by the European ESPAD survey, which found that 26.5% of 16-year-olds in Croatia have gambled in the past 12 months (in equal proportions via the internet and at land-based outlets), with Croatia ranking eighth in terms of gambling prevalence among adolescents compared to all 33 European countries that participated in the survey [39].

A part of the answer that explains such high rates lies in the fact that about twenty years ago, Croatia transitioned from closed and relatively monopolistic regulation of all gambling products to a very liberal gambling market, which impacted the rapid expansion of land-based gambling operators [40]. In addition, in recent years, there has been a significant increase in online gambling opportunities and increased availability of self-service betting terminals in bars [3,13,25,41] that are available to minors despite formal legal prohibition [3].

Presented trends led to a need for the development of the full range of interventions for adolescents, with prevention as one of the most important [18,42,43] and cost-effective [44] ways to address the problem of adolescent gambling and target individuals who may not yet be involved in this behaviour but are part of an at-risk group [20,21,27,45,46,47,48,49].

Preventive interventions can be divided into three main categories: (1) primary/universal (activities aimed at the adolescent population in general, regardless of identified risk or need, to prevent the development of gambling problems and raise awareness of the risks and consequences), (2) secondary/indicated (interventions for children at risk to reduce the likelihood of developing severe problem gambling behaviour) and (3) tertiary/selective (targeting adolescents who are already showing signs and symptoms of problem gambling and mainly involving treatment programs) interventions [27,44]. The literature has shown that universal school-based prevention is the preferred choice for problems of a social and behavioural nature in adolescence, as it has the advantage of both improving adolescents’ skills and reducing their internalising (e.g., anxiety, somatic symptoms, etc. [50,51]) and externalising (e.g., disruptive conduct, aggressive behaviour, etc. [50,51]) problems [18,27,36,44]. St-Pierre and Derevensky [43] classified school-based gambling-specific prevention programs into two categories: (1) psychoeducational prevention programs and (2) comprehensive prevention programs that include psychoeducation and skills training. Regardless of the category, prevention programs generally share key areas that influence the success of interventions. These are increasing awareness and knowledge about gambling and gambling problems, including the nature of gambling, the probabilities and chances of gambling, erroneous cognitions and gambling fallacies, the warning signs of gambling, and the possible negative psychosocial consequences. The second group of prevention programs additionally focuses on socio-emotional skills such as self-esteem, interpersonal and coping skills, problem-solving skills, decision making, and peer refusal skills [18,27,36,44,52,53].

Although school-based programs are an essential component of an overall strategy to prevent problem gambling [27], the number of such programs worldwide is still insufficient, and there is an increased need for evaluation research on their effectiveness [2,27,54,55,56,57,58]. Many of the prevention programs tend to have generic topics and their method of implementation depends on the decision of the professionals providing the program, while in most cases, their effectiveness is not verified [2,27,42,53,57,58]. When it comes to adherence to evidence-based principles, such as participant needs, scope, different teaching methods, clarity and measurability of goals set, appropriateness (in terms of age, culture, etc.) and sufficient duration, training of program leaders (program providers), and regular evaluation of effectiveness [59,60,61] of the existing programs, only few (e.g., the video program “Lucky”, “Count Me On”, “Kids Don’t Gamble... Wanna Bet?”, “Don’t Gamble Away Our Future”, “Too Much Too Loose” [18,55,62,63]) are structured and in line with these fundamental principles (e.g., the programs of Ferland, Ladouceur, and Vitaro, [52]; Williams, Wood, and Currie, [53]; Calado et al. [27]; Tani et al. [44]). In addition, there appears to be a lack of published scientific work explaining the development and implementation of such prevention programs with subsequent evaluation; thus, there is limited evidence of their effectiveness and insight into potential outcomes. The potential iatrogenic effects are equally insufficiently measured [36,43,57].

Moreover, in Croatia, a country with alarming rates of youth problem gambling and a great need for preventive interventions, no gambling-specific prevention programs existed until recently. To respond to these challenges, an interdisciplinary team of experts with diverse expertise (prevention, adolescent risk behaviours, cognitive psychology, mathematics of gambling, gambling addiction) developed, piloted, and evaluated a comprehensive universal school-based youth gambling prevention program called “Who Really Wins?” [36]. The program was piloted in two high schools in the city of Zagreb (n = 190 first and second grade students), with the authors of the program as implementors (program leaders). An experimental design with two groups (training vs. no training) and two measurement sessions (pre- and post-test) was used. Results showed significant effects on gambling knowledge and gambling-related cognitive distortions in the post-test sessions in the training group, while no changes were observed in the control group. Direct effects on protective factors such as problem-solving skills, resisting peer pressure skills, and general self-efficacy were not found. The results also confirmed the effects of the program regardless of school type, gender, and learning aptitudes, supporting the universality of the program. In addition, the program had no iatrogenic effects on behaviour change [36].

After the pilot implementation and evaluation of the aforementioned program, structural modification was performed. Initially, the program consisted of six 90-min workshops (2 school hours), but such a structure required extensive organisational modifications in the regular high school schedule. Therefore, the program was carefully modified into nine 45-min workshops with the same outcomes, making it easier to implement in the high school curricula. When the program was finalised, the authors created a comprehensive 3-day (21-h) training with the program package that consists of the implementation manual for future program leaders, workbooks for students, and pre- and post-test evaluation questionnaires. The Croatian Ministry of Science and Education, together with the Education and Teacher Training Agency, approved and supported the program, making it available as a prevention activity for high schools across Croatia. Up to this point, seven training sessions were conducted with school professionals, after which they could implement the program and administer the evaluation questionnaires. Thus, the purpose of this study is to examine the short-term effectiveness of this comprehensive universal youth gambling prevention program, “Who Really Wins?”, conducted by the trained program leaders, representing its first national evaluation. It also aims to contribute to a body of scientific evidence on the effectiveness of different prevention interventions in a school setting and to overcome previous shortcomings in terms of relatively small samples [58].

## 2. Materials and Methods

### 2.1. Participants

The study comprised n = 629 high school students from 18 Croatian towns/cities in different regions (66.5% males (n = 418), 33.5% females (n = 211); mean age = 15.67 years, SD = 0.73). Most of the students were enrolled in 4-year vocational programs (66.8%), followed by gymnasium programs (21.3%), while the lowest number of students attended 3-year vocational programs (11.9%). The study was conducted during school hours since the program was implemented as part of the regular prevention curricula in schools. Since program leaders were not obliged to conduct the evaluation, it is expected that in some schools, the program was implemented without administering and/or submitting evaluation forms. Therefore, the information on the proportion of submitted forms in overall participants of the program is not available; the sample is convenient and includes only students who finished the program and whose evaluation questionnaires were sent to the authors.

### 2.2. Measures

An evaluation questionnaire is an integral part of the program package and consists of a comprehensive battery of instruments.

Gambling Behaviour.

The past two-month frequency of gambling was assessed for five different gambling activities (sports betting, virtual race betting, slot machines, lottery, and scratch cards), as Croatian research confirms that these are the most frequently played games among high school students [64,65]. Gambling behaviour was assessed in the pre-test and post-test sessions, with students indicating how often they participated in specific games of chance (0 = not once (did not gamble); 1 = once or twice; 2 = about 1–2 times a month; 3 = about once a week; 4 = a few times a week; 5 = almost every day).

Gambling-Related Knowledge [36].

Knowledge about gambling was assessed using 20 statements constructed specifically for the evaluation of this program. For each statement, a student could answer “true”, “false”, or “do not know” (e.g., “Lotto and scratch tickets are games of chance, but not gambling”). The content covers different areas of knowledge (types of gambling, odds and probabilities, consequences of gambling). An overall score is calculated as the measure of proportion of correct answers, with a higher score indicating better gambling-related knowledge (more correct answers). In the pre-test, the test had an average difficulty for students of *p* = 0.56 (range of 0.08 to 0.94), while in the post-test, the overall difficulty was easier (*p* = 0.68), which is to be expected after the intervention.

Gambling-Related Cognitive Distortions [65].

Cognitive distortions related to gambling were assessed using the Gambling-Related Cognitive Beliefs Scale [65], which was previously validated in Croatian studies with high school students [13,64,66]. The questionnaire consists of two factors: (1) illusion of control based on knowledge and skills (5 items, e.g., “It is possible to predict the outcomes of gambling”) and (2) probabilistic reasoning and superstition (9 items, e.g., “A person can feel when he or she will be lucky in gambling”). Factor analysis confirmed the expected two-factor solution both in the pre-test (KMO = 0.915; Bartlett’s test of sphericity χ^2^ = 2828.39; *p* < 0.001) and the post-test (KMO = 0.942; Bartlett’s test of sphericity χ^2^ = 4236.97; *p* < 0.001), explaining 48.62% of total variance in the pre-test and 57.12% in the post-test. Internal consistency of the first and second factors is α = 0.78, α = 0.85 in the pre-test, respectively, and α = 0.80, α = 0.90 in the post-test, respectively. More detailed information about gender specific reliability can be found in Table 2.

Problem Solving Skills [36].

Problem-solving skills were assessed using a 5-item scale constructed specifically to evaluate the effectiveness of this prevention program. For each item, students had to answer how they normally handle problem situations in life (e.g., “When solving problems, I think about the pros and cons of possible solutions”). Responses were given on a five-point scale, from never to almost always. In a previous (pilot) study, the scale showed satisfactory reliability [36], with the one-dimensional solution explaining 46% of the total variance in the pre-test and 58% of the total variance in the post-test. In this study, the unidimensional solution was confirmed to explain 48.84% of the total variance before and 56.50% after the intervention. Internal consistency in this sample is adequate (α = 0.70 in the pre-test and α = 0.81 in the post-test; see Table 2 for more details on gender-specific results). Higher scores indicate better problem-solving skills.

Resisting Peer Pressure Skills [36].

Peer pressure resisting skills were assessed using a brief 5-item scale constructed specifically to evaluate the effectiveness of this program. For each item, students had to answer how they normally handle peer pressure situations (e.g., “I can easily tell when others are putting pressure on me.”). Responses were given on a five-point scale from “never” to “almost always”, with a higher score indicating better skills. In the previous (pilot) study, this scale showed appropriate metric properties and a unidimensional factor solution that explained 36% of the total variance in the pre-test and 58% in the post-test [36]. However, in this study, one item had low saturation in the first factor and low negative correlation with other items. Therefore, the internal consistency of this scale in this sample is poor (α = 0.42 on the pre-test and α = 0.65 on the post-test; see Table 2), so the results are discussed with caution, highlighting the need to use a more adequate instrument to measure this construct.

General Self-Efficacy [67].

Self-efficacy was assessed using the Generalised Self-Efficacy Scale [67]. This widely used scale has 10 items and measures one’s general sense of perceived self-efficacy and belief that one can master new or difficult tasks or cope with adversity in various areas of human functioning (e.g., “I always manage to solve difficult problems if I try hard enough”). Responses for each item ranged from 1 (strongly disagree) to 5 (strongly agree). The composite score is calculated as the average score for all items, with a higher score indicating higher perceived generalised self-efficacy. In this study, the scale had a high internal consistency of α = 0.86 at pre-test and α = 0.90 at post-test (see Table 2 for gender-specific information).

Problem Gambling Severity Scale (GPSS) [68].

The severity of problem gambling among adolescents was assessed using the General Problem Severity Subscale (GPSS) of the Canadian Adolescent Gambling Inventory (CAGI; [68]). This instrument has been validated with Croatian adolescents in previous national prevalence studies [13,66]. Based on the calculated results, adolescents are classified into three risk categories: (1) green light (no gambling-related problems; 0–1 points); (2) yellow light (low to moderate gambling-related problems; 2–5 points); and (3) red light (high severity of gambling-related problems; 6+ points). Participants indicated on a four-point scale (0 = never; 1 = sometimes; 2 = most of the time; 3 = almost always) how often they felt a particular consequence of their gambling activities (e.g., “How often have you felt bad about the way you gamble/bet or what happens when you gamble/bet?”; “How often have you gone back another day to try to win back the money you lost while gambling/betting?”). The scale has good internal consistency (α = 0.79 at pre-test and α = 0.89 at post-test, with more detailed information in Table 2).

### 2.3. Procedure, Intervention and Evaluation Design

This study examines the short-term effectiveness of the youth gambling prevention program “Who Really Wins?”, implemented by trained school psychosocial professionals (school counsellors) and teachers. Pre- and post-tests were administered as part of the program protocol. A pre-test was administered at the beginning of the first workshop (introduction and motivation for the program), while the post-test was administered after the last workshop. The program consists of 9 workshops with students, typically over 9 weeks (once a week for 45 min), a two-hour interactive lecture with parents of youth involved in the program, and a two-hour interactive lecture with all school staff. In general, the program falls into the second group of school-based prevention programs according to St-Pierre and Derevensky [43], as it addresses socioemotional skills in addition to psychoeducational components. A more detailed description of the topics within the workshops can be found in the Table 1. The interactive lectures for parents and school staff covered various topics with the aim of better understanding the gambling industry in Croatia, highlighting individual and social risk factors for adolescent gambling, learning about the prevalence and consequences of adolescent gambling, and being able to recognise early symptoms. The lectures were interactive, meaning that all participants were encouraged to ask questions and discuss their thoughts and possible experiences. These lectures were not evaluated.

The program is based on empirical evidence on predictors of gambling involvement and gambling-related problems [13,25,36,38,64,66,69,70] and already established effective youth gambling prevention efforts [36], and is consistent with the evidence-based principles mentioned above [71]. It is aimed at adolescents aged 14 to 16 and was developed in several phases from 2012 to 2015.

The overall aim of this program is to prevent and/or delay participation in gambling activities and to contribute to personally responsible behaviour in this area. Accordingly, the specific objectives relate to reducing false gambling beliefs (illusion of control, superstitious thinking, and probabilistic reasoning) and improving knowledge about gambling, as well as socio-emotional skills that are crucial for the prevention of risky behaviour in general (problem-solving skills, resistance to peer pressure, critical thinking, assertiveness, etc.) [71]. To ensure adequate national implementation and dissemination of the program, the authors published a manual and created a three-day training (21 h) for future program leaders, while a specific workbook was created for the students. The entire program package, which includes anonymous evaluation questionnaires, was accepted and approved by the Croatian Ministry of Science and Education and the Education and Teacher Training Agency. The program is to be implemented by two leaders, one of whom must be a school counsellor (social pedagogue, psychologist, or pedagogue) and the other can be a teacher (of any course). To date, seven training sessions have been conducted and more than 200 teachers and school counsellors across the country have completed the training.

Since the evaluation is an integral part of the program and the regular school-based prevention curriculum, no specific student consent was obtained. The evaluation questionnaires were anonymous and coded, with each participant given a special key on how to create a personal code. In this way, researchers were able to pair pre- and post-test questionnaires with complete anonymity for students. Furthermore, the participants of the program could decide not to fill in the questionnaires and to opt out at any point. The program leaders collected the questionnaires in their schools and sent them by mail to the researchers at the University of Zagreb. Questionnaires collected in the period from 2017 to 2020 were included in the analysis for the purposes of this study.

### 2.4. Data Analysis

The first step was to examine skewness and kurtosis in pre-test and post-test scores for all continuous variables whose changes were to be assessed, for the entire sample and separately by gender (Table 2). These included variables related to knowledge, cognitive distortions, problem-solving skills, and ability to resist peer pressure, as well as general self-efficacy and measures of problem gambling. The results showed that parametric analysis was appropriate for all variables [72,73,74], except for the measure of problem gambling (GPSS total score). Therefore, a paired-samples *t*-test with the effect size was conducted for all variables, except for the frequency of gambling (ordinal scale) and GPSS total score, where a non-parametric Wilcoxon rank sum test was conducted to examine differences between the pre-test and post-test. A Bonferroni correction was used for multiple comparisons, so the original significance of *p* < 0.05 is set on *p* < 0.001.

## 3. Results

This prevention program is universal and, therefore, suitable for all high school students, regardless of recognised risk or need. Nevertheless, in order to have a clear picture of the participants and to explore possible iatrogenic effects in the area of gambling behaviour, we assessed the frequency of gambling in the last two months (Appendix A). Due to its high accessibility, availability, and attractiveness, and according to previous research, sports betting is the most frequently played gambling game among Croatian adolescents [13,29,75]. The results of this study confirm previous findings. At the pre-test, 9.5% of all students (14% of boys) regularly bet on sports results (once a week or more), and about 12% of them (17.6% of boys) bet occasionally.

In terms of gambling-related problems (Appendix B), the CAGI-GPSS results indicate that about 7% of all students participating in the program (about 9% of boys and 2% of girls) already developed a high severity of adverse gambling-related psychosocial consequences (red light), while a low to moderate severity of consequences (yellow light) was found among 13% of students (about 18% of boys and 3% of girls). These results suggest that a different range of interventions is possibly needed for some students.

Short-term intervention effects are presented in Table 3 and Table 4. Results for all students showed medium positive effects on cognitive variables after the program in terms of increased gambling-related knowledge, less illusion of control, superstition, and incorrect concepts of probability related to gambling. Small or trivial effects were found on gambling behaviour in the past two months on two games of chance (sports betting and lottery), but overall, mean scores for frequency of gambling are relatively low. Results suggest that the program did not have a significant impact on students’ socioemotional skills, but mean scores for these variables were relatively high in the pre-test.

The results for a subsample of boys are similar, which is to be expected given that they comprise 66% of the total sample. The results for girls did not show effects on gambling behaviour, but their gambling was not so prevalent in the pre-test. An important feature of these results is the fact that all effects are obtained in the desired direction (higher levels of correct knowledge, lower levels of cognitive distortions, lower frequency of gambling in two games of chance), proving that there are no iatrogenic effects on the measured variables.

Another important aspect of testing possible iatrogenic effects is the level of problem gambling, i.e., the severity of negative psychosocial consequences. The Wilcoxon rank test did not show significant changes between the pre- and post-test, additionally supporting the program’s effectiveness.

## 4. Discussion and Study Limitations

Short-term effectiveness evaluation of the universal prevention program “Who Really Wins?” suggests that the program achieves its purpose in gambling prevention. The results show that it is effective in reducing gambling-related cognitive distortions and improving gambling knowledge, while no effects on socio-emotional skills were observed.

The latter is consistent with the findings of an earlier pilot evaluation of this program [36] and with the evaluation of other prevention programs targeting risk behaviours [43,76], where similar results were found. Explanations of these trends could be twofold. First, it is not recommended to measure socioemotional skills immediately after the intervention [43,77,78,79,80], as it takes time for behaviour change to be visible and measurable [81,82,83]. Second, even though high school students already have developed capacity for self-reflection, their self-report in assessing socioemotional competencies can be sensitive to different biases: (1) memory effects (they may not accurately recall behaviours or actions), (2) social desirability bias (e.g., respondents may provide answers they think are “correct” instead of answers that represent their actions/beliefs), and (3) reference biases (e.g., their self-ratings are influenced by the competencies of others with whom they interact) [80,84]. Therefore, the most suitable assessment methods for measuring inter- and intrapersonal skills are those that allow students to demonstrate their ability to use their socioemotional skills in an applied setting (e.g., observation and/or rubric assessments of performance in group projects) since they are increasingly valuable for determining whether their skills may be transferable to settings outside the classroom environment [84].

When it comes to cognitive distortions, they are continuously confirmed as significant predictors of problem gambling and as a fundamental part of problem gambling symptomatology [70,85,86,87,88,89,90]. Consequently, approaches that challenge cognitive distortions and provide educational corrections have shown success in the treatment of problem gambling [85,91]. When it comes to prevention, research by Donati et al. [26] showed that cognitive distortions have a mediating role in the relationship that links probabilistic reasoning fallacy and superstitious thinking with youth problem gambling and that they can be efficiently impacted through prevention strategies, which is corroborated by the findings of this study, i.e., by a significant decrease in illusion of control, superstitious beliefs, and incorrect understanding of probability. In terms of positive effects on gambling knowledge, the trend is also consistent with both the pilot study of this program [36] and with research in this area, indicating that incorrect knowledge is an important mechanism underlying risky behaviour [92,93,94]. On the other hand, there is also evidence of dissociation between knowledge and behaviour change [95,96], suggesting that focusing on knowledge alone is insufficient in terms of prevention efforts. Accordingly, one of the fundamental postulates of prevention science states that effective programs are those that combine knowledge and skill-based activities [60]. Therefore, despite the fact that effects on socioemotional skills were not measured in the short-term evaluation of this program, there is no doubt that the focus should be on enhancing socio-emotional skills, especially those that have been shown to be important in preventing risky behaviour, such as problem-solving and refusal skills, critical thinking, etc. [18,27,36,44,52,53].

In addition, this study confirmed the findings of a previous pilot study that the program had no iatrogenic effects on the measured variables. More specifically, the frequency of gambling stayed low and even reduced for two games of chance, while gambling-related problems were low in both sessions with no significant change. This result is encouraging and confirms the program’s effectiveness, considering that one of the risks of creating a program aimed at preventing risky behaviour is precisely the potentially harmful impact—primarily behavioural effects—in that it arouses curiosity in participants and encourages them to participate in risky behaviour/consumption [59].

The cumulative findings of this study are important for several reasons. First, the program shows promising results as being an effective tool in youth gambling prevention with potential for its further dissemination. It is evidence-based, created following quality standards of psychosocial interventions [60], and developed in several phases by an interdisciplinary team of experts. In addition, it has been continuously evaluated, whereas a significant proportion of prevention programs in this field lack evidence-based foundations and evaluations, as stated in the introductory chapter. Even when an evaluation is conducted, it is predominantly either an evaluation of a preventive activity (e.g., a psychoeducational lecture) or a relatively small sample of participants is included in the evaluation research [2,27,43,58]. Thus, this program and its evaluation study represent an important contribution to research on intervention effectiveness.

In addition, research on adolescent gambling in Croatia consistently confirms that gambling is a major public health problem, which underlies the need for developing a full range of interventions for adolescents, with an emphasis on prevention. For this reason, this universal prevention program adequately addresses the at-risk population and responds to current problems, which is supported by the fact that it has been recognised by the relevant national institutions that regulate education processes and curricula.

However, the ongoing and systematic implementation and evaluation of the program also raise some questions and challenges. First of all, there is the question of whether it is really a universal program, knowing that gambling among young people in Croatia is a predominantly male phenomenon, i.e., girls participate in gambling and develop gambling-related problems to a much lesser extent [13,25,36]. On the other hand, if we move away from the Croatian context, where the rates of adolescent problem gambling and gambling participation are extremely high, we see that the rates of problem gambling among girls are not so insignificant in terms of foreign indicators [97,98,99,100,101]. Furthermore, the results of this short-term evaluation show effects on cognitive variables in the subsample of girls. Thus, it can be concluded that this program, although predominantly focused on the “male problem”, is effective for all students, regardless of gender. However, in the long term, there remains a question about the possible need to develop a gender-specific intervention. As well, the baseline data confirmed that a significant proportion of high school students have developed problem gambling despite their young age and that up to 20% of participants manifest some level of gambling-related consequences. Therefore, the question arises whether they are adequate participants for such a program, i.e., whether it is necessary to involve them in treatment interventions, instead. However, an argument could be made that since the program focuses extensively on aspects important for the prevention of risky behaviour, but also for behaviour change (e.g., knowledge about gambling and harmful consequences, cognitive distortions, socio-emotional skills), this group of adolescents also benefits from it. There is no doubt, however, that this hypothesis should be explored in future studies and that efforts must also be made in developing treatment interventions.

Another possible limitation of this research is the fact that the program was implemented by numerous (previously trained) leaders, which means that the method of implementation is not completely uniform, especially considering the different initial vocation and competencies of program leaders. However, a prerequisite for the implementation of the program is comprehensive training so that all measures are taken to ensure program fidelity since it is a conditio sine qua non in ensuring the reliability and validity of evaluation studies [102]. In addition, the program leaders provided their feedback on conducting each workshop (the overall impression, timeframe, competencies, strengths, and limitations of each workshop, etc.), and this aspect will be addressed in future studies focusing on process evaluation results.

Methodological shortcomings include that this study design, compared to the pilot study, did not comprise a control group [36]. Nevertheless, based on the results of the pilot study, it can be indirectly concluded that the effects of the program can be attributed to the intervention. There is also a lack of evidence on long-term effectiveness, which is necessary to determine the lasting effects of the program, especially given the behavioural changes that are difficult to capture with a paper-pencil method and take time to be acquired. More specifically, a change in cognition alone (both knowledge and cognitive distortions), especially when tested directly after the program, does not tell us whether these changes will positively affect future behaviour. Moreover, since gambling participation was measured immediately after the program, i.e., for the period during which students participated in the workshops, the results should be taken with caution. Therefore, these results must be considered preliminary and a basis for the next research design that will attempt to overcome these shortcomings with the goal of more adequately measuring the impact of the program on these important constructs.

Finally, given the context in which the program is implemented, it is questionable whether a universal prevention program can achieve and sustain its positive effects in an environment where gambling is extremely available, accessible, and socially accepted. It is, therefore, crucial to invest not only in interventions that target young people, but also in improving gambling legislation. Only with such a comprehensive approach can young people be protected as one of the most vulnerable groups to develop gambling-related problems.

## 5. Conclusions

Although it is not without limitations, this study offers evidence that the “Who Really Wins?” program is a promising and useful and tool in youth gambling prevention. The findings highlight that the impacts of the program are most apparent in terms of gambling-related knowledge and cognitive distortions, while effects on socio-emotional skills are limited and indicate the need for the evaluation of long-term effects.

Created on the foundation of youth gambling research and principles of evidence-based interventions, “Who Really Wins?” is a solid example of a developmentally appropriate prevention program that shows promise for promoting children’s socio-emotional learning, suppressing early gambling participation in order to reduce the chances of future involvement and problem gambling.

Moreover, given the lack of evaluation studies of psychosocial interventions in the area of adolescent gambling, this study makes an important contribution to knowledge in this field. The presented results also implicate the need for further research on interventions effectiveness, with a goal of overcoming the shortcomings in methodology and implementation and reaching a consensus on “what works” when it comes to youth gambling prevention.

## Figures and Tables

**Table 1:** Structure and the goals (themes) of the workshops within the youth gambling prevention program “Who Really Wins?”.

No.	Title	Goals (Themes)
0	Introductory (+pre-test)	Introducing the implementation of the prevention program, motivating students for participation, and administrating pre-test questionnaires.
1	What do we need to know about the Program?	Introducing the topics (themes) of the prevention program and setting the rules of the group work.
2	What is the other side of the medal?	Introducing students with the main characteristics and consequences of gambling (and problem gambling) and placing them in perspective with other youth risk behaviours.
3	What to do when the dice is thrown?	Introducing students with the consequences of gambling through their active participation in workshop activities and encouraging critical thinking.
4	What are my chances?	Teaching students about general concepts of odds and probability in the games of chance, randomness, and independence of events, and facilitating correlation of these concepts with possible stakes, risks, and rewards.
5	I have a problem, what is my choice?	Teaching students about problem solving skills, enabling the practice of these skills in the workshop, and facilitating critical thinking about choices and consequences.
6	How to be a part of the group and stay myself?	Teaching students about skills related to resisting peer pressure and self-advocacy and enabling the practice of these skills in the workshop.
7	In the end—who really wins?	Facilitating introspection (insights) and wrapping key content (themes), gained knowledge and skills.
8	Did we learn in all?	Wrapping the process through feedback, certification of students and evaluation of the process. Administration of the post-test questionnaire.

**Table 2:** Skewness, kurtosis and Cronbach’s alpha reliability for all variables (all sample- and gender-specific).

	All Sample (n = 629)			Boys (n = 418)			Girls (n = 211)		
	Skew.	Kurt.	α	Skew.	Kurt.	α	Skew.	Kurt.	α
Gambling-related knowledge (T1)	−0.56	0.26	n.a.	−0.55	0.27	n.a.	−0.61	0.13	n.a.
Gambling-related knowledge (T2)	−1.28	1.82	n.a.	−1.30	1.87	n.a.	−1.21	1.58	n.a.
Illusion of control (T1)	−0.12	−0.47	0.78	0.01	−0.50	0.78	−0.40	−0.27	0.77
Superstition and incorr. prob.(T1)	0.73	0.31	0.85	0.79	0.59	0.85	0.61	−0.28	0.85
Illusion of control (T2)	0.38	−0.36	0.80	0.45	−0.30	0.80	0.25	−0.47	0.79
Superstition and incorr. prob.(T1)	1.26	1.24	0.90	1.32	1.41	0.91	1.13	0.90	0.90
Problem solving skills (T1)	−0.36	0.15	0.70	−0.34	0.08	0.70	−0.40	0.35	0.69
Peer pressure skills (T1)	−0.26	0.52	0.42	−0.36	1.03	0.41	−0.08	−0.41	0.44
Problem solving skills (T2)	−0.49	0.34	0.81	−0.44	0.25	0.82	−0.46	0.24	0.77
Peer pressure skills (T2)	−0.46	0.86	0.65	−0.51	0.82	0.69	−0.15	−0.08	0.54
General self-efficacy (T1)	−0.65	1.61	0.86	−0.76	2.01	0.86	−0.46	1.10	0.85
General self-efficacy (T2)	−0.74	1.49	0.90	−0.77	1.33	0.91	−0.46	0.91	0.86
Total GPSS (T1)	3.21	12.42	0.79	2.62	8.27	0.78	7.47	64.94	0.80
Total GPSS (T2)	4.47	25.37	0.89	3.85	18.88	0.89	7.59	67.89	0.88

Legend: Skew. = skewness; Kurt. = kurtosis; α = Cronbach’s alpha.

**Table 3:** Mean scores compared with paired-samples *t*-tests at pre- and post-test (short-term effectiveness).

	Variables (Theoretical Range)	Pre-Test (T1)		Post-Test (T2)		T	d
		M	SD	M	SD		
**All sample** **(n = 629)**	Knowledge (0–20)	11.28	3.58	13.62	4.02	−14.63 *	−0.58
	Illusion of Control (1–5)	2.77	0.89	2.32	0.89	12.15 *	0.48
	Superstition and incorr. prob. (1–5)	1.96	0.73	1.70	0.75	9.14 *	0.36
	Problem solving skills (1–5)	3.74	0.67	3.75	0.75	−0.28 (n.s.)	−
	Peer pressure skills (1–5)	3.47	0.57	3.51	0.69	−1.12 (n.s.)	−
	General self-efficacy (1–5)	3.76	0.63	3.75	0.71	0.36 (n.s.)	−
**Boys (n = 418)**	Knowledge (0–20)	11.36	3.73	13.48	4.12	−10.57 *	−0.52
	Illusion of Control (1–5)	2.72	0.92	2.30	0.92	9.52 *	0.47
	Superstition and incorr. prob. (1–5)	1.95	0.74	1.69	0.76	7.46 *	0.36
	Problem solving skills (1–5)	3.74	0.67	3.69	0.79	1.20 (n.s.)	−
	Peer pressure skills (1–5)	3.49	0.58	3.49	0.74	(n.s.)	−
	General self-efficacy (1–5)	3.80	0.63	3.73	0.77	1.96 (n.s.)	−
**Girls (n = 211)**	Knowledge (0–20)	11.12	3.27	13.89	3.82	−10.60 *	−0.73
	Illusion of Control (1–5)	2.86	0.83	2.39	0.86	7.56 *	0.52
	Superstition and incorr. prob. (1–5)	1.99	0.71	1.73	0.72	5.26 *	0.36
	Problem solving skills (1–5)	3.76	0.66	3.87	0.66	−2.71 (n.s.)	−
	Peer pressure skills (1–5)	3.44	0.57	3.54	0.58	−2.22 (n.s.)	−
	General self-efficacy (1–5)	3.67	0.62	3.77	0.60	−2.56 (n.s.)	−

Legend: n.s. non-significant; * *p* < 0.001 (criteria based on Bonferroni correction).

**Table 4:** Wilcoxon rank test on gambling behaviour and the total CAGI-GPSS score.

		Pre-Test (T1)		Post-Test (T2)		Mean Rank		Z	
		M	SD	M	SD	Neg. R.	Pos. R.		
**All sample (n = 629)**	Gambling—sports betting (0–5)	0.53	1.20	0.38	0.95	77.98	59.58	−4.41 *	0.18
	Gambling—virtual races (0–5)	0.27	0.88	0.18	0.69	44.64	44.22	−2.87 (n.s.)	
	Gambling—slot machines (0–5)	0.19	0.78	0.12	0.55	29.67	34.17	−2.42 (n.s.)	
	Gambling—lotto (0–5)	0.25	0.83	0.13	0.57	43.25	38.52	−4.15 *	0.17
	Gambling—scratch cards (0–5)	0.19	0.72	0.14	0.58	42.42	42.63	−2.15 (n.s.)	
	Gambling—roulette (0–5)	0.10	0.58	0.10	0.56	18.00	20.06	−0.15 (n.s.)	
**Boys (n = 418)**	Gambling—sports betting (0–5)	0.76	1.38	0.54	1.09	71.57	56.36	−4.47 *	0.22
	Gambling—virtual races (0–5)	0.38	1.03	0.26	0.81	40.80	39.91	−2.65 (n.s.)	
	Gambling—slot machines (0–5)	0.26	0.92	0.16	0.63	27.59	28.91	−2.38 (n.s.)	
	Gambling—lotto (0–5)	0.32	0.94	0.16	0.64	36.80	30.60	−3.62 *	0.18
	Gambling—scratch cards (0–5)	0.21	0.78	0.17	0.67	29.49	31.13	−1.33 (n.s.)	
	Gambling—roulette (0–5)	0.14	0.79	0.14	0.63	17.18	16.81	−0.84 (n.s.)	
**Girls (n = 211)**	Gambling—sports betting (0–5)	0.06	0.47	0.06	0.40	7.00	4.50	−0.05 (n.s.)	
	Gambling—virtual races (0–5)	0.05	0.38	0.03	0.30	4.50	4.50	−1.41 (n.s.)	
	Gambling—slot machines (0–5)	0.05	0.34	0.04	0.31	3.00	6.00	−1.00 (n.s.)	
	Gambling—lotto (0–5)	0.12	0.55	0.06	0.36	7.29	8.75	−2.30 (n.s.)	
	Gambling—scratch cards (0–5)	0.16	0.57	0.09	0.35	13.50	10.50	−2.03 (n.s.)	
	Gambling—roulette (0–5)	0.01	0.15	0.04	0.40	1.50	3.50	−0.74 (n.s.)	
**Total** **GPSS**	All (n = 629)	1.14	2.57	1.17	3.2	104.06	120.73	−1.23 (n.s.)	
	Boys (n = 418)	1.59	2.93	1.58	3.67	89.04	103.86	−1.45 (n.s.)	
	Girls (n = 211)	0.27	1.25	0.35	1.76	15.13	17.88	−0.42 (n.s.)	

Legend: n.s. non-significant; * *p* < 0.001 (criteria based on Bonferroni correction).

## Data Availability

The data presented in this study are available on request from the corresponding author. The data are not publicly available due to privacy issues.

## Appendix A

**Table A1:** Frequency of gambling in the past 2 months (pre-test and post-test).

		Pre-Test (T1) %						Post-Test (T2) %					
Type of Game	Sample	0	1	2	3	4	5	0	1	2	3	4	5
Sports betting	All	78.3	8.9	3.2	3.5	3.8	2.2	81.6	8.9	4.1	2.6	1.8	0.9
	Boys	68.5	12.9	4.7	5.4	5.6	3.0	73.8	12.1	6.1	4.0	2.8	1.2
	Girls	97.3	1.3	0.4	0.0	0.4	0.4	96.4	2.7	0.4	0.0	0.0	0.4
Virtual races	All	88.9	4.3	2.9	1.1	1.4	1.4	91.2	4.6	1.1	1.7	1.1	0.3
	Boys	84.8	5.1	4.4	1.6	2.1	1.9	87.6	6.3	1.6	2.6	1.4	0.5
	Girls	96.9	2.7	0.0	0.0	0.0	0.4	98.2	1.3	0.0	0.0	0.4	0.0
Slot machines	All	91.6	4.0	1.7	0.6	0.8	1.4	94.5	2.3	1.8	0.6	0.5	0.3
	Boys	88.8	5.1	2.1	0.9	0.9	2.1	92.5	3.0	2.8	0.5	0.7	0.5
	Girls	96.9	1.8	0.9	0.0	0.4	0.0	98.2	0.9	0.0	0.9	0.0	0.0
Lotto	All	88.3	4.8	3.1	1.5	1.4	0.9	93.5	3.1	1.7	0.9	0.5	0.3
	Boys	86.2	5.1	4.0	1.6	1.6	1.4	91.8	4.2	1.9	0.9	0.7	0.5
	Girls	92.4	4.0	1.3	1.3	0.9	0.0	96.9	0.9	1.3	0.9	0.0	0.0
Scratch cards	All	89.4	6.1	1.8	0.9	0.8	0.9	91.9	5.1	1.4	0.8	0.6	0.3
	Boys	89.5	6.1	1.4	0.9	0.7	1.4	91.6	4.4	1.6	0.9	0.9	0.5
	Girls	89.2	6.3	2.7	0.9	0.9	0.0	92.4	6.3	0.9	0.4	0.0	0.0
Roulette	All	96.0	1.8	0.6	0.3	0.5	0.8	95.4	2.3	0.6	0.6	0.6	0.5
	Boys	94.4	2.6	0.7	0.5	0.7	1.2	93.7	3.3	0.9	0.9	0.5	0.7
	Girls	99.1	0.4	0.4	0.0	0.0	0.0	98.7	0.4	0.0	0.0	0.9	0.0

Legend: 0 = not once (did not gamble); 1 = once or twice; 2 = about 1–2 times a month; 3 = about once a week; 4 = a few times a week; 5 = (almost) every day.

## Appendix B

**Table A2:** Prevalence of students’ problem gambling (CAGI-GPSS).

GPSS Categorisation	All (%) n = 629		Boys (%) n = 418		Girls (%) n = 2110	
	Pre-Test (T1)	Post-Test (T2)	Pre-Test (T1)	Post-Test (T2)	Pre-Test (T1)	Post-Test (T2)
Green light	80.0	83.0	72.2	76.8	95.3	95.3
Yellow light	13.4	10.0	18.4	13.9	3.3	2.4
Red light	6.7	7.0	9.3	9.3	1.4	2.4

Legend: Green light: no problems; Yellow light: low-to-moderate severity of adverse psychosocial consequences; Red light: high severity of adverse psychosocial consequences.
